# Early Worsening of Diabetic Retinopathy Following Initiation of Hybrid Closed‐Loop/Automated Insulin Delivery Systems in Type 1 Diabetes: A Systematic Review and Structured Study‐Level Synthesis

**DOI:** 10.1111/dom.71084

**Published:** 2026-07-08

**Authors:** Matthew Anson, Ahmed Iqbal, Shazli Azmi, Philip Weston, Lucy Hughes, Mahreen Choudhury, David M. Hughes, Yalin Zheng, Philip I. Burgess, Uazman Alam

**Affiliations:** ^1^ Diabetes & Endocrinology Research, Institute of Life Course and Medical Sciences University of Liverpool Liverpool UK; ^2^ Department of Diabetes & Endocrinology University Hospitals of Liverpool Group Liverpool UK; ^3^ School of Medicine and Population Health The University of Sheffield Sheffield UK; ^4^ Division of Diabetes, Endocrinology and Gastroenterology, Faculty of Biology, Medicine and Health University of Manchester Manchester UK; ^5^ Sheffield Medical School The University of Sheffield Sheffield UK; ^6^ Department of Health Data Science, Institute of Population Health University of Liverpool Liverpool UK; ^7^ Department of Eye and Vision Sciences, Institute of Life Course and Medical Sciences University of Liverpool Liverpool UK; ^8^ St Pauls Eye Unit University Hospitals of Liverpool Liverpool UK; ^9^ Centre for Biomechanics and Rehabilitation Technologies Staffordshire University Stoke‐on‐Trent UK

**Keywords:** diabetic retinopathy, insulin pump therapy, systematic review, type 1 diabetes

## Abstract

**Background:**

Hybrid closed‐loop (HCL) systems achieve rapid, algorithm‐driven improvements in glycaemia in type 1 diabetes (T1D). Paradoxically, rapid improvement in glycaemic control is associated with early worsening of diabetic retinopathy (EWDR), a phenomenon established in the intensive insulin therapy era. Whether HCL initiation carries a clinically meaningful EWDR risk is unknown. No systematic review has previously addressed this question.

**Methods:**

A systematic review and structured quantitative synthesis was performed using study‐level estimates only (PROSPERO CRD:420261391951). MEDLINE, SCOPUS and Web of Science were searched to 14th May 2026. Studies reporting retinal outcomes in people with T1D initiating any HCL system were eligible. Two reviewers independently screened studies and extracted data. Risk of bias was assessed using ROBINS‐I and certainty of evidence using the GRADE framework. EWDR incidence was summarised using study‐level proportions, and comparative studies were summarised using study‐specific risk ratios for HCL versus control therapy. Given substantial heterogeneity in EWDR definitions, retinal assessment timing, follow‐up duration, and comparator groups, no pooled or meta‐analytic estimates were derived.

**Results:**

Eight studies (*n* = 1487 participants; 860 HCL users) were included; all were observational and six were retrospective. EWDR varied markedly with the timing of retinal assessment. In studies assessing the retina within ≤ 12 months of HCL initiation, EWDR rates ranged from 8.9% to 26.5%. Studies with longer follow‐up reported lower rates of retinal worsening or incident DR, 6.7% at 24 months and 6.1% over a mean follow‐up of 4.9 years, suggesting that these studies may capture background DR progression rather than true early worsening. Three comparative studies included 177 HCL users and 315 controls; EWDR study‐specific risk ratios were directionally inconsistent, ranging from 0.32 to 1.51, and were therefore not pooled. The most consistently identified risk factors were higher baseline HbA1c and older age. The magnitude of HbA1c reduction was not a consistent predictor of EWDR in the HCL context, in contrast to pre‐HCL era evidence. Risk of bias ranged from moderate to critical and certainty of evidence was very low for all outcomes.

**Conclusions:**

Study‐defined retinal worsening was reported in a minority of participants. The current evidence base is dominated by retrospective studies, variable retinal assessment timing, and inconsistent EWDR definitions. Well‐designed prospective studies with protocol‐specified retinal surveillance anchored to HCL initiation are required to generate reliable incidence estimates, identify risk factors, determine visual consequences, and inform standardised screening guidance.

## 1 Introduction

Diabetic retinopathy (DR) remains the leading cause of preventable vision loss in people of working age, affecting more than one‐third of individuals with diabetes globally and carrying a lifetime risk exceeding 90% in type 1 diabetes (T1D) [1]. Despite advances in ophthalmic treatment, DR continues to impose significant morbidity, with sight‐threatening complications, including proliferative DR and diabetic macular oedema [2]. The primary determinant of DR risk and progression is chronic hyperglycaemia, and the long‐term benefit of optimised glycaemic control in preventing and slowing DR is unequivocal, established by the Diabetes Control and Complications Trial (DCCT) and confirmed across decades of subsequent evidence [3].

However, the relationship between glycaemic improvement and DR is not linear in the short term. A well‐characterised paradox exists: rapid improvement in glycaemic control, while conferring long‐term retinal protection, is associated with early worsening of diabetic retinopathy (EWDR), a transient deterioration in retinal status occurring within the first 3–12 months of glycaemic intensification [4]. First systematically characterised in the DCCT, EWDR was observed in 13.1% of intensively treated participants within 6–12 months, compared with 7.6% in the conventional therapy group [5]. Subsequent systematic reviews have confirmed EWDR incidence of 10%–20% across diverse intensive treatment contexts, including insulin pump therapy, GLP‐1 receptor agonist initiation, and bariatric surgery [6, 7]. While EWDR is often transient and reversible over 12–24 months in those with minimal baseline DR, it can lead to irreversible retinal damage in patients with pre‐existing moderate‐to‐severe non‐proliferative or proliferative DR.

Hybrid closed‐loop (HCL) systems, also termed Automated insulin delivery (AID) systems, represent the most transformative advance in insulin delivery technology since the development of continuous subcutaneous insulin infusion. By integrating continuous glucose monitoring with an insulin pump governed by a real‐time dosing algorithm, HCL systems achieve substantial and rapid improvements in time‐in‐range (TIR), glycaemic variability, and HbA1c, with benefits demonstrated in randomised trials [8] and real‐world data across the age spectrum [9, 10]. Since first regulatory approval in 2016, multiple commercial systems are in widespread and rapidly expanding use [11]. The glycaemic gains achieved by HCL are often rapid: real‐world cohorts have demonstrated HbA1c reductions of 1.7% within the first 6 months of initiation [9], particularly in those with suboptimal prior control, precisely the group at highest risk of EWDR.

No randomised controlled trial to date has included retinal outcomes as a prespecified endpoint in a HCL cohort, and many pivotal HCL trials have explicitly excluded patients with advanced DR. Current guidance from the American Diabetes Association recommends retinal assessment when glucose‐lowering therapy is intensified [12], but no specific ophthalmological surveillance protocol exists for HCL initiation.

This review aimed to synthesise studies reporting objectively assessed retinal outcomes in people with T1D initiating or using HCL therapy. The specific objectives are to describe the reported frequency of study‐defined EWDR among HCL‐exposed participants; summarise comparative estimates versus non‐HCL controls where available; identify reported risk factors for EWDR after HCL initiation; and characterise the timing, grading methods and retinal monitoring protocols used across studies.

## 2 Methods

### 2.1 Protocol and Registration

This systematic review was conducted and reported in accordance with the Preferred Reporting Items for Systematic Reviews and Meta‐Analyses (PRISMA) 2020 statement. The protocol was registered prospectively on PROSPERO prior to data extraction (CRD:420261391951).

### 2.2 Eligibility Criteria

Papers that report on people of any age with a confirmed diagnosis of T1D who initiated any commercial or investigational HCL insulin delivery system. No restriction on age, sex, ethnicity, diabetes duration, prior insulin delivery modality, or geographic setting.

Any HCL system providing automated basal and/or correction insulin delivery guided by continuous glucose monitoring. This includes but is not limited to: Tandem t:slim X2 with Control‐IQ; Medtronic MiniMed 780G; Insulet Omnipod 5; CamAPS FX; open‐source systems (Loop, AndroidAPS, iAPS). Sensor‐augmented pump therapy with predictive low glucose suspend alone (no automated correction bolus) was classified as a comparator, not an intervention.

Studies with or without a comparator group were eligible. Comparators included: (i) pre‐HCL within‐person baseline DR status (before‐after); (ii) multiple daily injections (MDI) with or without CGM; (iii) sensor‐augmented pump without automated correction.

Primary outcome: incidence or prevalence of EWDR, defined as any worsening in retinal grade after HCL initiation, assessed by validated retinopathy grading or progression to treatment (laser or intravitreal agents). Studies with longer follow‐up were included in the broader synthesis but analysed separately or explored in sensitivity analyses because they may capture longer‐term DR progression rather than EWDR. Secondary outcomes: (i) magnitude of DR worsening; (ii) reversibility at 12–24 months; (iii) risk factors for EWDR; (iv) sight‐threatening events within 12 months; (v) monitoring protocols and (vi) vision.

RCTs, prospective cohort studies, retrospective cohort studies, and before‐after observational studies were eligible. No language restriction was applied; non‐English full texts were translated where necessary.

### 2.3 Exclusion Criteria

Studies in T2D only; studies in pregnant women; case reports (< 5 participants); studies without objective retinal outcome data (self‐report only).

### 2.4 Search Strategy

A comprehensive search was conducted in MEDLINE (via Ovid), SCOPUS and Web of Science Core Collection from inception to 14th May 2026. Grey literature was sought via conference abstract archives (ATTD, EASD, ADA, 2016 to date). The full search strategy is provided in Supporting Information.

### 2.5 Study Selection

All search results were imported into Rayyan (an open‐access, free electronic reference manager) and deduplicated. Title and abstract screening were performed independently by two reviewers (M.A. and M.C.) against pre‐defined eligibility criteria using a standardised screening form. Any disagreements were resolved by consensus and consultation with another senior author. Full‐text review of potentially eligible articles was performed independently by both reviewers. Disagreements at any stage were resolved by discussion; unresolved disagreements were adjudicated by a third senior reviewer. A PRISMA 2020 [13] flow diagram was produced.

### 2.6 Data Extraction

Data extracted included author, year, country, study design, sample size, age, sex, diabetes duration, baseline HbA1c, baseline retinopathy status, HCL system, comparator type, retinal grading method, follow‐up duration, EWDR definition, number of events, treatment‐requiring events, HbA1c change, CGM metrics, and reported risk factors. Data extraction was performed by one reviewer using a standardised extraction form and verified by another co‐reviewer.

### 2.7 Risk of Bias and Certainty of Evidence

Risk of bias was assessed independently by two reviewers, with disagreements resolved through discussion and adjudication by a third reviewer where required. Bias was assessed using ROBINS‐I [14], considering bias due to confounding, participant selection, classification of interventions/exposures, deviations from intended interventions, missing data, outcome measurement, and selection of the reported result. Studies were not excluded on the basis of risk of bias, but assessments were incorporated into the interpretation of findings and certainty of evidence. Certainty of evidence was appraised using the GRADE (Grading of Recommendations, Assessment, Development and Evaluations) framework. Because all included studies were observational, the initial certainty rating for each outcome was low. Five domains were evaluated for potential downgrading: risk of bias, inconsistency, indirectness, imprecision, and publication bias. Three upgrade factors (large effect, dose–response gradient, direction of plausible confounding) were considered but did not apply. Certainty was assessed independently for three outcomes: (i) EWDR incidence in HCL users; (ii) risk factors for EWDR and (iii) treatment requiring DR.

### 2.8 Quantitative Synthesis

Quantitative synthesis was conducted in R using the meta package.

Given substantial clinical and methodological heterogeneity across studies, including differences in EWDR definitions, retinal imaging and grading methods, retinal assessment timing, follow‐up duration and comparator groups, no pooled or meta‐analytic summary estimates were derived. Instead, a structured quantitative synthesis was undertaken descriptively. For single‐arm cohorts, study‐specific EWDR proportions were calculated using the number of participants with EWDR as the numerator and the number with gradable follow‐up retinal assessment as the denominator. For comparative cohorts, study‐specific risk ratios were calculated for HCL versus control therapy, with controls comprising MDI or open‐loop CSII as defined by each study. Exact binomial 95% confidence intervals were calculated for single‐arm proportions, and 95% confidence intervals were calculated for risk ratios. All study‐level estimates were displayed in forest plots without pooled summary diamonds.

## 3 Results

### 3.1 Overview

**FIGURE 1:**
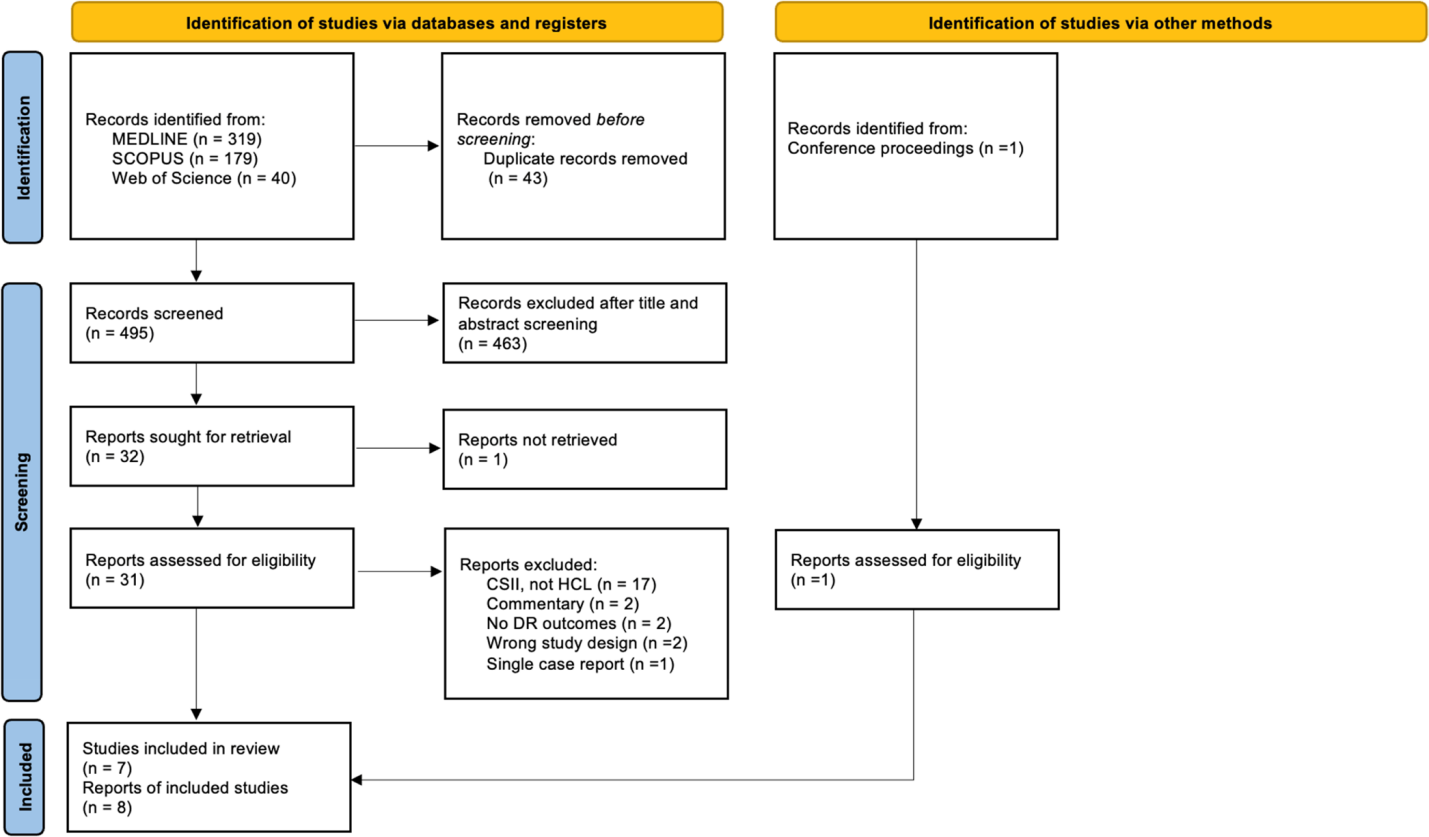
PRISMA 2020 flowchart.

The database searches retrieved 538 records, and after deduplication 495 records remained. Following title and abstract screening, 31 full‐text articles were assessed for eligibility, of which 24 were excluded (most commonly: ‘open loop’ CSII [*n* = 17]; commentaries [*n* = 2]; retinal outcome data not reported [*n* = 2]; inadequate study design or case report [*n* = 2] and a single case report [*n* = 1]). Searching of conference proceedings identified [*n* = 1] additional study not retrieved by database searches. Eight studies met full eligibility criteria and were included in the review (seven in the quantitative synthesis). Details are shown in Figure 1 (PRISMA 2020 flow diagram).

The eight included studies were published between 2023 and 2026, representing a recently emerging body of literature coinciding with widespread adoption of HCL systems in clinical practice. Six were retrospective observational studies and two (Nattero‐Chávez et al. [15] and Barquiel et al. [16]) were prospective studies. Only three studies included a comparator group: Diddhenipothage et al. [17] compared HCL against open‐loop continuous subcutaneous insulin infusion (CSII), while Nattero‐Chávez et al. [15] and Barquiel et al. [16] each compared HCL against multiple daily injections (MDI). The remaining five studies were single‐arm cohorts [10, 18, 19, 20, 21]. Collectively, 1487 individuals with T1D were included across all studies, of whom 860 used HCL therapy. The HCL system used varied across studies; the majority used either the Medtronic MiniMed 780G or the Tandem t:slim X2 with Control‐IQ. Baseline patient demographics and study characteristics are shown in Tables 1 and 2, respectively.

**TABLE 1:** Baseline patient characteristics.

Author, year, location	N, baseline demographics	Baseline DR
Diddhenipothage [17], 2024, UK	*N* = 148 HCL group (*n* = 74) 64% Female Mean duration of T1D 28 ± 13 years Open loop CSII (*n* = 74) 55% Female Mean duration of T1D 27 ± 15 years	HCL group R0: 22/74; R1: 24/74; R2: 5/74; R3S: 7/74; R3A: 3/74; M0: 48/74; M1: 13/74; Missing data: 13/74 Open loop CSII R0: 21/74; R1: 31/74; R2: 2/74; R3S: 2/74; R3A: 6/74; M0: 56/74; M1: 6/74 Missing data: 12/74
Nattero‐Chávez [15], 2026, Spain	*N* = 379 HCL group (*n* = 115) 53% Female; Mean age 38 ± 13 years Mean duration of T1D 24 ± 11 years MDI group (*n* = 264) 41% Female; Mean age 42 ± 13 years Mean duration of T1D 25 ± 12 years	HCL group No DR: 85.2%; Mild NPDR: 6.1%; Moderate NPDR: 3.5%; Severe NPDR: 4.3%; Proliferative DR: 0.9%; Any DR: 15% (*n* = 17) MDI group No DR: 86.4%; Mild NPDR: 6.4%; Moderate NPDR: 3.0%; Severe NPDR: 3.0%; Proliferative DR: 1.1%; Any DR: 13.6% (*n* = 36)
Karakus [19], 2025, USA	*N* = 152 57% Female; Mean age 42 ± 14 years Mean duration of T1D 26 ± 13 years	Baseline ETDRS score Rt and Lt eye: 27 ± 16
Barquiel [16], 2025, Spain	*N* = 60 HCL group (*n* = 30) 70% Female; Mean age 48 ± 8 years Mean duration of T1D 34 ± 9 years MDI group (*n* = 30) 30% Female; Mean age 48 ± 8 years Mean duration of T1D 33 ± 10 years	HCL group Mild NPDR: 63.3%; Moderate NPDR: 13.3%; Severe NPDR: 3.3%; Proliferative DR: 20% MDI group Mild NPDR: 43.3%; Moderate NPDR: 3.3%; Severe NPDR: 3.3%; Proliferative DR: 50%
Liarakos [10], 2025, UK	*N* = 420 68% Female Median age 40 (IQR 29–50) Median duration of T1D 21 years (15–29 years)	Not reported
Johansson [21], 2025, New Zealand & Australia	*N* = 165 59% Female; Mean age 28.4 ± 15.5 years Mean duration of T1D 15.7 ± 10.3 years	Nil (R0/M0): 46%; Minimal (M1/R1): 12%; Mild (M2/M3/R2): 26%; Moderate (M4/R3): 3%; Severe (M5/R4): 1%; Proliferate (R5): 1%; Previously treated: 12%
Andrew [18], 2023, UK	*N* = 62 68.1% Female; Mean age 40.3 ± 12.0 years Mean duration of T1D 23.3 ± 11.1 years	Baseline retinopathy present in 20 (32.2%) individuals
Ung [20], 2025, France	*N* = 101 60% Female; Mean age 21 ± 3 years Mean duration of T1D 12 ± 5 years	Not reported

**TABLE 2:** Baseline study characteristics.

Author, year, location	Study design	HCL system/control	Baseline HbA1c/glucometrics	Follow up	DR grading method	Retinopathy as primary outcome?
Diddhenipothage [17], 2024, UK	Retrospective study	HCL group 77% Tandem t:slim X2 with Control‐IQ; 21.6% Medtronic MiniMed 780G; 1.3% CamAPS FX Open loop CSII Not stated	HCL group HbA1c: 62 ± 17 mmol/mol Open loop CSII HbA1c: 69 ± 17 mmol/mol	12‐18 months	NDESP R0‐3 and M0‐1	Yes
Nattero‐Chávez [15], 2026, Spain	Prospective cohort	100% Medtronic MiniMed 780G Control: 100% MDI	HbA1c: 57.4 ± 10.9 mmol/mol TIR not reported for group as a whole	Mean follow up: 4.9 ± 1.4 years	ICDR severity scale	Yes
Karakus [19], 2025, USA	Retrospective study	100% Tandem t:slim X2 with Control‐IQ No control group	HbA1c: 7.6% ± 1.3%	2.7 years; DR graded anytime from 3 months to > 2 years	Qualitative and ETDRS	Yes
Barquiel [16], 2025, Spain	Prospective observational	100% Medtronic MiniMed 780G Control: 100% MDI	Not stated	2 years	Qualitative	Yes
Liarakos [10], 2025, UK	Retrospective study	47% Medtronic MiniMed 780G 36% Tandem t:slim X2 with Control‐IQ 4% CamAPS FX 4% Medtrum; 2% Medtronic MiniMed 670G; 7% Not recorded No control group	HbA1c: 79 ± 10 mmol/mol TIR 34% ± 14%	6–38 months	Not stated	No
Johansson [21], 2025, New Zealand & Australia	Retrospective study	50% Medtronic MiniMed 780G 27% Tandem t:slim X2 with Control‐IQ; 23% OpenAPS No control group	HbA1c: 68.8 ± 17.6 mmol/mol TIR 34% ± 14%	Mean time to follow up grading 8.7 ± 7.2 months	NZ National Diabetes Retinal Grading System and International Clinical Disease Severity Scale/ICDR	Yes
Andrew [18], 2023, UK	Retrospective study	Not stated No control group	HbA1c: 76.4 ± 12.4 mmol/mol TIR not reported for group as a whole	3 months	NDESP R0‐3 and M0‐1	Yes
Ung [20], 2025, France	Retrospective study	91.1% Tandem t:slim X2 with Control‐IQ; 6.9% Medtronic MiniMed 780G; 2% Diabeloop No control group	HbA1c: 82 ± 25 mmol/mol TIR 39% ± 16%	12 months	Not stated	No

### 3.2 EWDR Definition

**FIGURE 2:**
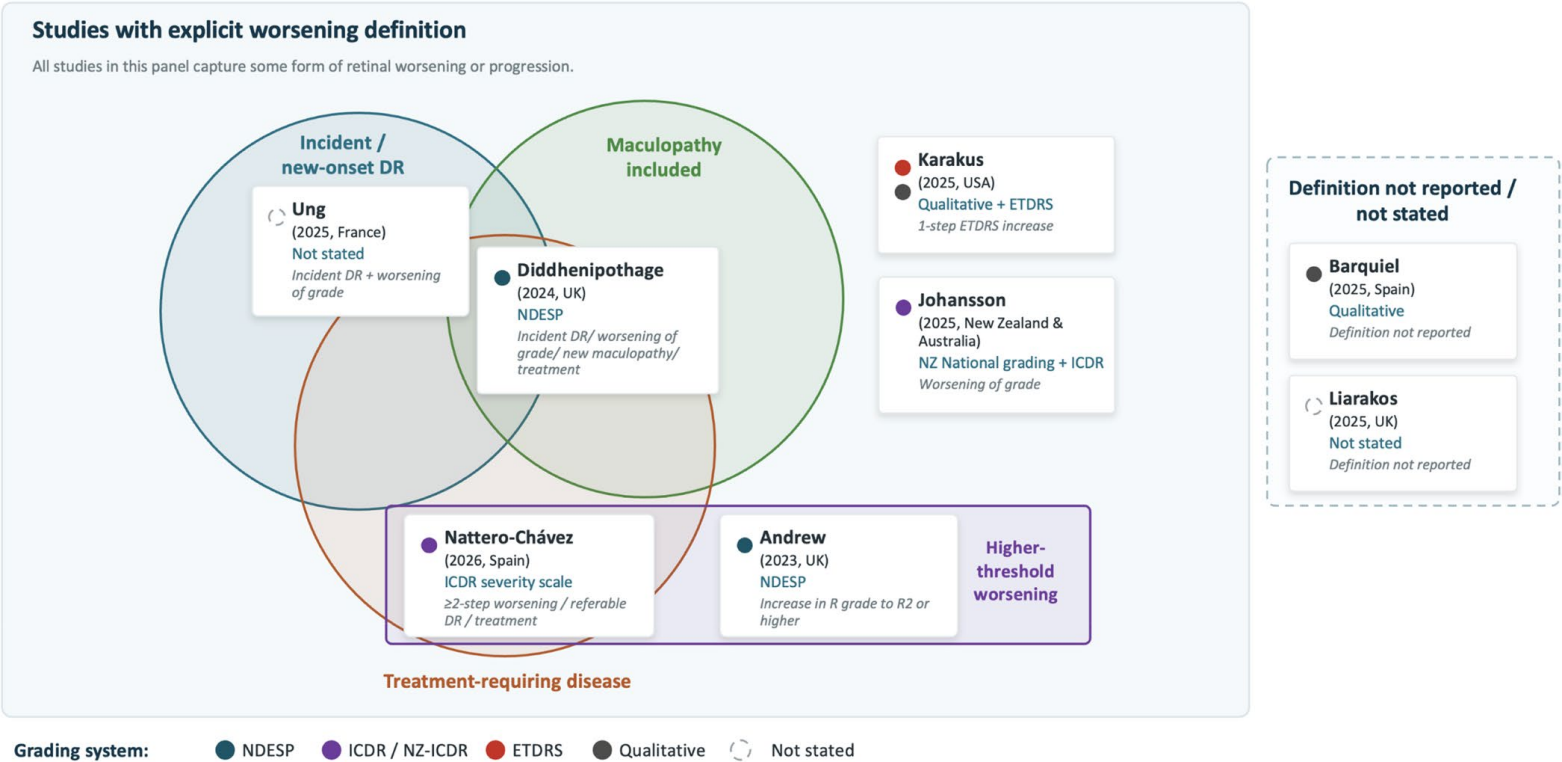
Heterogeneity of EWDR definitions across included studies.

A critical observation across all included studies was the heterogeneity of both retinal outcome definitions, timing of retinal assessment and grading system used, which substantially limits direct comparability. No two studies used an identical definition of EWDR. Diddhenipothage et al. [17] defined EWDR broadly to include incident DR, worsening of any existing DR grade, development of maculopathy, or initiation of treatment, a composite that captures a range of clinical events spanning from subclinical changes to sight‐threatening complications. Johansson et al. [21] applied a more granular approach, separately grading retinopathy (R) and maculopathy (M) grade changes, defining worsening as any increase in either grade whilst Andrew et al. [18] require an increase in R grading to R2 or higher as part of the UK NDESP to be classified as an EWDR event. Karakus et al. [19] used both ETDRS scores and qualitative clinical assessment, while Barquiel et al. [16] used a qualitative grading system whose details were not fully reported. Nattero‐Chávez et al. [15] applied the ICDR severity scale with a two‐step progression threshold for disease progression. Ung et al. [20] reported new‐onset DR and worsening of pre‐existing DR as separate events without combining them into a single composite. In two studies (Liarakos et al. [10] and Ung et al. [20]), retinopathy was not a primary endpoint, and no formal EWDR definition was applied. The heterogeneity in outcome definitions across studies is summarised in Figure 2.

### 3.3 Timing of Follow Up Fundal Screening

The timing of retinal assessment relative to HCL initiation also varied substantially. Andrew et al. [18] assessed retinal status at a fixed 3‐month follow‐up, which represents the shortest post‐initiation window of any included study and is well‐aligned with the expected peak EWDR window from the DCCT literature. At the other extreme, Karakus et al. [19] graded retinopathy at any point between 3 months and more than 2 years after HCL initiation, a window so broad that it is difficult to attribute observed changes specifically to the period of early glycaemic improvement rather than natural longitudinal DR progression. Johansson et al. [21] had a mean time to follow‐up grading of 8.7 months (SD 7.2 months), indicating wide within‐study variation in assessment timing. Nattero‐Chávez et al. [15] had a mean follow‐up of 4.9 years, with retinal assessments at annual intervals; as discussed further below, this study's design is better suited to evaluating longer‐term DR incidence and progression than specifically characterising EWDR in the immediate post‐initiation period. These methodological differences mean that, in practice, the included evidence base conflates EWDR, the short‐term paradoxical phenomenon of interest, with background longitudinal DR progression, and this conflation cannot be fully resolved through sensitivity analysis alone.

### 3.4 EWDR Results

**FIGURE 3:**
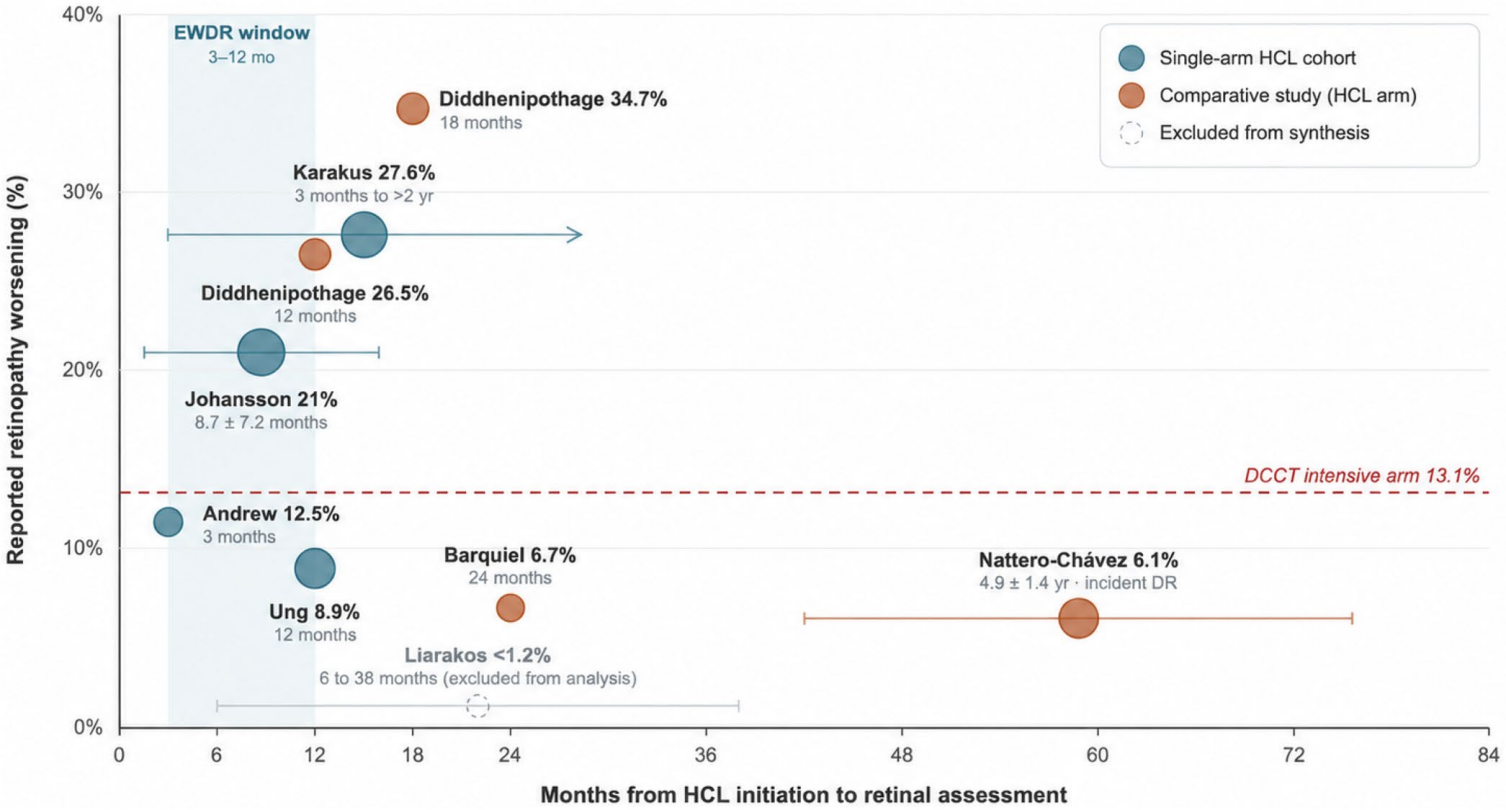
Reported EWDR by timing of retinal assessment after HCL initiation. Each study is positioned by the timing of retinal assessment (*x*‐axis) and its reported worsening rate (*y*‐axis); circles are proportional to the number of HCL participants assessed. The shaded band denotes the 3–12‐month EWDR window and the dashed line the DCCT intensive‐arm rate (13.1%). Horizontal bars indicate mean ± SD assessment/follow‐up time (Johansson, Nattero‐Chávez), the reported open‐ended assessment window (Karakus), or the follow‐up range (Liarakos). Johansson et al. rate shown is 21% (34/165, R‐ or M‐grade worsening); Nattero‐Chávez et al. includes reported incident DR.

Individual study‐level EWDR rates are summarised in Table 3. Across the six studies that reported a quantifiable EWDR rate in HCL users, proportions ranged from 8.9% (Ung et al. [20], composite of new‐onset and worsening DR) to 28% (Karakus et al. [19]). The lowest rate, reported by Nattero‐Chávez et al. [15] (6.1% incident DR in the HCL arm), reflects a fundamentally different outcome metric, newly detected DR in a predominantly DR‐free cohort over nearly 5 years, rather than short‐term EWDR, and should not be compared directly with the other figures. The higher rates reported by Diddhenipothage et al. [17] (26.5% within 12 months in the HCL arm) and Karakus et al. [19] (28%) likely reflect both genuine early worsening and background progression captured over longer or uncontrolled follow‐up windows. Two studies (Liarakos et al. [10] and Andrew et al. [18]) reported notably lower figures, in part because of their short follow‐up or the fact that retinopathy was not a primary endpoint with fewer than five events reported [10]. The relationship between assessment timing and reported worsening across studies is shown in Figure 3 and Table 4.

**TABLE 3:** EWDR incidence by study.

Author, year, location	n/N with EWDR	EWDR detail	Definition used
Diddhenipothage [17], 2024, UK	HCL group: 12 months: 13/49 (26.5%); 18 months: 17/49 (34.7%). Open loop CSII: 12 months: 10/57 (17.5%); 18 months: 13/57 (22.8%)	≥ 2% (22 mmol/mol) drop in HbA1c at 12 m associated with highest rate of EWDR	Patients with R3AM0/R3S excluded from follow up. EWDR defined as either: development of new DR, worsening of DR grade in either eye, development of maculopathy or need for treatment
Nattero‐Chávez [15], 2026, Spain	HCL group incident DR 6/98 (6.1%, 95% CI: 2.8–12.7) MDI group incident DR 43/228 (18.9%, 95% CI: 14.3–24.4)	HCL use was associated with a 67.2% reduction in DR risk vs. MDI (HR = 0.328, 95% CI: 0.126–0.726; *p* = 0.01). Among 53 participants with DR at baseline, 15 (28.3%) experienced progression. All progression events occurred in the MDI group	Incident DR defined as the first detection of any DR among participants free of DR at baseline. Progression defined as: (1) worsening of 2 or more steps in ICDR scale, (2) progression to referable DR (≥ moderate NPDR and/or DME), or (3) initiation of a DR‐specific intervention.
Karakus [19], 2025, USA	42/152 (28%) had DR worsening. Most of these progressions were from no DR to mild non‐proliferative DR.	Baseline HbA_1c_ was significantly higher in the DR worsening group compared with no‐DR worsening group (*P* = 0.02). Participants with DR worsening had higher HbA_1c_ after HCL initiation (7.7 ± 1.5 vs. 7.0 ± 0.9; *p* = 0.003). In mixed models, no significant change in ETDRS score in either eye by time interval.	DR worsening was defined as an increase in Early Treatment of Diabetic Retinopathy Study (ETDRS) scores or qualitative retinal exam
Barquiel [16], 2025, Spain	HCL group: 2/30 (6.7%) progressed MDI group: 4/30 (13.3%) progressed. Between group difference (*p* = 0.389)	Remission of NPDR occurred in *n* = 15 (50%) treated with HCL compared with none in the MDI group (*p* < 0.001). Stage improvement was observed in *n* = 15 (50%) of HCL users and *n* = 2 (6.7%) of MDI users (*p* < 0.001)	Not reported
Liarakos [10], 2025, UK	Worsening of retinopathy *n* < 5 (< 1.2%)	Worsening of retinopathy occurred in individuals with a high baseline Hba1c (> 9%) and up to 1.4% reduction in HbA1c at follow‐up.	Not reported
Johansson [21], 2025, New Zealand & Australia	21% worsened R or M grade. R grade: 26/145 (18%) worsened; 25/145 (17%) improved; 94/145 (65%) no change. M grade: 16/145 (11%) worsened; 19/145 (13%); improved; 110/145 (76%) no change	In individuals with a HbA1c change > 1.5% vs. ≤ 1.5%, progression of R grade RR 1.02 (0.39‐2.17). In individuals with a HbA1c change > 1.5% vs. ≤ 1.5%, progression of M grade RR 0.94 (0.70‐1.26)	Improvement defined as any decrease in either R or M grade (or both), stability as no change in either retinopathy or maculopathy grade, and worsening as any rise in either R or M grade (or both).
Andrew [18], 2023, UK	In completed follow‐up (*n* = 32), 4 (12.5%) people had progression of retinopathy (*n* = 2 new pre‐proliferative, *n* = 2 proliferative requiring treatment). No new maculopathy was observed.	Among those who progressed, no difference was noted in baseline HbA1c (progression 75.7 vs. 76.4 mmol/mol; *p* = 0.91) or TIR (progression 33.5% vs. 37.8%; *p* = 0.59). All individuals progressing to R2 or higher had retinopathy at baseline. No new maculopathy was observed.	Progression was defined as any increase in R grading to R2 or higher
Ung [20], 2025, France	5/101 (5%) new onset DR. 4/101 (4%) worsened their R grade, mostly by 1 stage	Not reported	Not reported

**TABLE 4:** Stratification by retinal assessment timing.

Time frame	Author, year, location	Follow up window	Reported EWDR rate
≤ 12 months	Andrew [18], 2023, UK	3 months	12.5% worsened
	Ung [20], 2025, France	12 months	5% new onset DR 4% worsened R grade
	Diddhenipothage [17], 2024, UK	12 months	26.5% worsened
Variable time window	Johansson [21], 2025, New Zealand & Australia	Mean 8.7 months, SD 7.2 months	21% worsened
	Karakus [19], 2025, USA	3 months to > 2 years	28% worsened
	Liarakos [10], 2025, UK	6–38 months	< 1.2% worsened
≥ 12 months	Nattero‐Chávez [15], 2026, Spain	Mean 4.9 years, SD 1.4 years	6.1% new onset DR No ‘worsening’
	Diddhenipothage [17], 2024, UK	18 months	34.7% worsened
	Barquiel [16], 2025, Spain	2 years	6.7% worsened

### 3.5 Clinically Meaningful Ophthalmic Outcomes

Overall, the granularity of reported ophthalmic treatment was poorly recorded, and no studies report visual acuity as an outcome. No studies commented on macular oedema as measured by optical coherence tomography, and only a small minority of individuals starting HCL required treatment with intravitreal therapies and/or photocoagulation, where such outcomes were reported (Table 5).

**TABLE 5:** Clinically meaningful reported ophthalmic outcomes.

Author, year, location	Clinically meaningful ophthalmic outcomes in HCL users
Diddhenipothage [17], 2024, UK	4/148 needed new DR treatment 1/148 needed further active DR treatment (baseline R33)
Nattero‐Chávez [15], 2026, Spain	All ophthalmic interventions: pan retinal photocoagulation, intravitreal therapy and vitrectomy occurred exclusively in MDI users only
Karakus [19], 2025, USA	After AID initiation: 14% reported to have proliferative retinopathy (not specified treatment) and 12% severe NPDR. Unclear reporting figure of PDR and NPDR at baseline.
Barquiel [16], 2025, Spain	Not reported
Liarakos [10], 2025, UK	Not reported
Johansson [21], 2025, New Zealand & Australia	5/165 progressed to require treatment. 4/165 worsened from non‐proliferative stage to a proliferative stage and 1/165 had new proliferation after previously treated proliferative disease
Andrew [18], 2023, UK	2/32 progressed to proliferative disease requiring treatment
Ung [20], 2025, France	Only 1 (< 1%) case of severe worsening of retinopathy that required laser treatment (occurring in a patient whose GMI decreased from 11.6% to 8.5% within 2 months of starting HCL, despite using exercise mode)

### 3.6 Risk Factors

Risk factors for EWDR are summarised in Table 6. The most consistently supported risk factors were higher baseline HbA1c and older age. Three studies identified higher baseline HbA1c as a significant predictor: Karakus et al. [19] reported a statistically significant association (mean HbA1c 8.0 vs. 7.4% in those with and without worsening respectively; *p* = 0.02) and Nattero‐Chávez et al. [15] demonstrated that each 1% increment in pre‐baseline HbA1c more than doubled the hazard of DR (HR 2.25, 95% CI 1.58–3.21). In contrast, Andrew et al. [18] found no difference in baseline HbA1c between those who did and did not progress, though this analysis was limited by very small numbers (*n* = 4 progressors). The relationship between the magnitude of HbA1c reduction and EWDR was inconsistent across studies. Only Diddhenipothage et al. [17] identified a clear signal, with a drop of ≥ 2% associated with the highest EWDR rate. Two studies found no association between the degree of HbA1c improvement and worsening (Johansson et al. [21], Karakus et al. [19]), which challenges the assumption that the magnitude of glycaemic improvement is the primary driver of EWDR in the HCL context, as established in the DCCT intensive insulin therapy era. Older age was identified as a significant independent predictor in two studies: Johansson et al. [21] reported an OR of 4.7 (95% CI 1.2–16.4) for age ≥ 18 years, and Nattero‐Chávez et al. [15] found a 4% increment in DR hazard per year of age (HR 1.04, 95% CI 1.01–1.07).

**TABLE 6:** Risk factors for EWDR.

Risk factor	Supporting and non‐supporting studies	Notes
Magnitude of HbA1c drop	2 Supporting 3 Non‐supporting	≥ 2% drop in HbA1c associated with higher prevalence of EWDR (*n* = 4) [17] Up to 1.4% reduction in HbA1c at follow‐up (*n* < 5) [10] No correlation of magnitude of HbA1c drop [19] Longitudinal increases in HbA1c during follow‐up were associated with greater DR risk (HR = 1.043 per 1% increase, 95% CI: 1.008–1.080; *p* = 0.02) [15] Rapid > 1.5% improvement in HbA1c not associated with DR worsening (OR 1.1, 95% CI 0.4‐3.4) [21]
Baseline HbA1c	3 Supporting 1 Non‐supporting	Higher baseline HbA1c was associated with a two‐fold increase in risk for DR worsening (OR 2.1, 95% CI 1.34–3.04) [19] Worsening of retinopathy occurred in individuals with a high baseline HbA1c (> 9%) [10] Each 1% increase in pre‐baseline HbA1c more than doubled the risk (HR 2.25, 95% CI 1.58–3.21) [15] Among those who progressed, no difference was noted in baseline HbA1c (progression 75.7 vs 76.4 mmol/mol; *p* = 0.91) or TIR (progression 33.5% vs. 37.8%; *p* = 0.59) [18]
Baseline DR severity	1 Supporting 1 Non‐supporting	All individuals progressing to R2 or higher had retinopathy at baseline [18] Moderate/severe DR at baseline was not associated with EWDR (OR 0.4, 95% CI 0.0–3.4) [21]
Age	2 Supporting 0 Non‐supporting	Older age was independently associated with increased DR risk (HR 1.04 per year, 95% CI 1.01–1.07) [15] Age at HCL initiation ≥ 18 years was a risk factor for any worsening of DR (OR 4.7, 95% CI 1.2–16.4) [21]
Diabetes duration	1 Supporting 1 Non‐supporting	One additional year of diabetes duration increased the hazard of DR by 7.3% (HR 1.07, 95% CI 1.04–1.11) [15] Duration of T1D > 15 years (OR 1.6, 95% CI 0.5–5.2) and > 10 years (OR 1.2, 95% CI 0.4–3.8) vs. < 10 years not associated with EWDR [21]

### 3.7 Quantitative Synthesis of Comparative Studies

Study‐specific risk ratios were directionally inconsistent, ranging from 0.32 to 1.51, and comparators included both MDI and open‐loop CSII. Given this heterogeneity, comparative findings were presented as study‐specific estimates without a pooled effect estimate.

Follow‐up duration varied substantially between studies, ranging from 12 to 59 months, with individual study follow‐up periods of 12 [17], 18 [17], 24 [16] and 59 [15] months. Across the included studies, EWDR occurred in 25/177 HCL users and 60 control participants.

**FIGURE 4:**
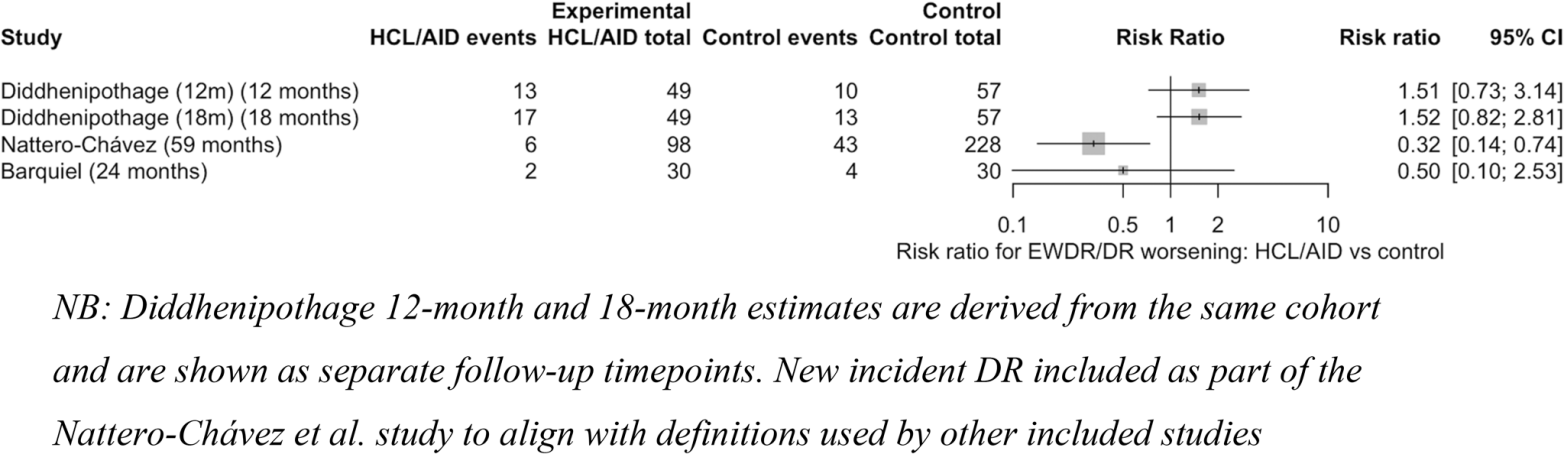
Study‐specific risk ratios for EWDR/DR worsening in HCL/AID versus control cohorts NB: Diddhenipothage 12‐month and 18‐month estimates are derived from the same cohort and are shown as separate follow‐up timepoints. New incident DR is included as part of the Nattero‐Chávez et al. study to align with definitions used by other included studies.

Nattero‐Chávez et al. [15] did not include incident DR within their study specific definition of EWDR. We have opted to include it within the quantitative synthesis to align with the definition of EWDR utilised by other studies. Diddhenipothage et al. [17] reported a numerically higher risk of EWDR among HCL users compared with open loop CSII over 12 months: 13/49 vs. 10/57, RR 1.51; 95% CI 0.73–3.14 and 18 months 17/49 vs. 13/57; RR 1.52; 95% CI 0.82–2.81. In contrast, Nattero‐Chávez et al. [15] reported a lower risk among HCL vs. MDI users: 6/98 vs. 43/228, RR 0.32; 95% CI 0.14–0.74. Barquiel et al. [16] also reported a numerically lower risk in the HCL group compared to MDI: 2/30 vs. 4/30, RR 0.50; 95% CI 0.10–2.53. (Figure 4).

### 3.8 Quantitative Synthesis of Single Arm Studies

Four studies of single arm cohorts comprising 430 individuals exposed to HCL therapy were included in the quantitative synthesis. Across these studies, 81 participants experienced EWDR or diabetic retinopathy worsening. Individual study event rates ranged from 8.9% to 27.6%. Because of this heterogeneity, results were presented as study‐level proportions without a pooled summary estimate.

**FIGURE 5:**
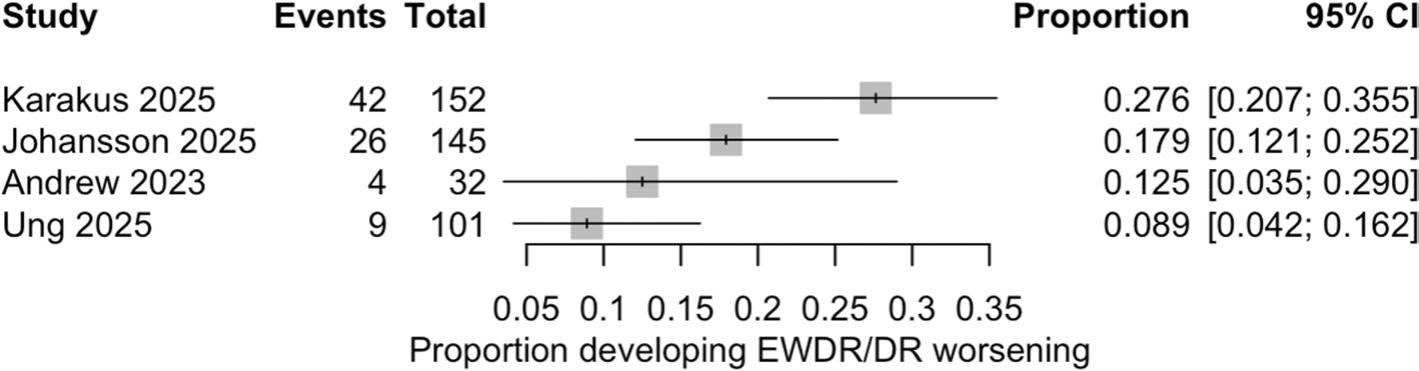
Study‐specific proportions of EWDR/DR worsening after HCL/AID therapy.

Johansson et al. [21] reported worsening of retinopathy (R) and maculopathy (M) grades separately; for consistency with the other included studies, we only included worsening of the R grade in the forest plot (Figure 5). For Ung et al. [20], we pooled both incident diabetic retinopathy and worsening of pre‐existing diabetic retinopathy to derive a composite retinopathy worsening outcome. Data from Liarakos et al. [10] were not included because insufficient numerical data were available to extract a compatible event rate.

### 3.9 Risk of Bias and Certainty of Evidence

Risk of bias was assessed using ROBINS‐I and is summarised in Table 7. Overall risk of bias was moderate in three studies, serious in three studies, and critical in two studies. Applying a GRADE‐based appraisal, the certainty of evidence was very low for all outcomes (Table 8). All included studies were observational and because confounding by baseline HbA1c, age, diabetes duration, baseline DR severity, prior treatment modality, and selection into HCL therapy cannot be excluded, no causal conclusion can be drawn regarding HCL initiation and EWDR risk, progression to clinically significant disease necessitating treatment, nor the risk factors associated with progression.

**TABLE 7:** Risk of bias assessment of included studies using ROBINS‐I.

Author, year, location	D1 confounding	D2 selection	D3 classification	D4 deviations	D5 missing data	D6 outcome measurement	D7 reporting	Overall ROBINS‐I
Diddhenipothage [17], 2024, UK	Serious	Serious	Low	Low	Critical	Moderate	Serious	Critical
Nattero‐Chávez [15], 2026, Spain	Moderate	Moderate	Low	Low	Low	Low	Low	Moderate
Karakus [19], 2025, USA	Moderate	Serious	Low	Low	Serious	Low	Moderate	Serious
Barquiel [16], 2025, Spain	Moderate	Moderate	Low	Moderate	Low	Low	Low	Moderate
Liarakos [10], 2025, UK	Moderate	Moderate	Low	Low	Critical	Critical	Low	Critical
Johansson [21], 2025, New Zealand & Australia	Moderate	Moderate	Low	Low	Moderate	Moderate	Moderate	Moderate
Andrew [18], 2023, UK	Serious	Serious	Low	Low	Serious	Moderate	Serious	Serious
Ung [20], 2025, France	Serious	Serious	Low	Moderate	Low	Moderate	Low	Serious

**TABLE 8:** GRADE certainty of evidence.

Outcome(s)	Reasons for downgrading certainty	Overall GRADE
EWDR incidence in HCL users	Serious risk of bias (retrospective design; ROBINS‐I moderate–critical; no protocol‐specified retinal surveillance) Serious inconsistency (rates 8.9%–27.6%; unexplained variation; cannot pool) Serious indirectness (non‐equivalent EWDR definitions; non‐comparable grading scales; assessment windows overlap EWDR period and natural progression)	**⊕**○○○ **VERY LOW**
Risk factors for EWDR	Serious risk of bias (retrospective extraction; limited multivariable adjustment; most studies underpowered for risk‐factor analysis) Serious inconsistency (HbA1c change: inconsistent; baseline HbA1c: 3 supporting, 1 non‐supporting; age: 2 supporting, 0 non‐supporting) Serious indirectness (different outcome definitions used as dependent variable; different HCL systems; predominantly European/Australasian) Suspected publication bias (within‐study selective reporting of positive predictors)	**⊕**○○○ **VERY LOW**
Treatment requiring DR	Serious risk of bias (not pre‐specified; no systematic ophthalmological protocol) Not assessable for inconsistency (*n* = 13 events total across studies) Serious indirectness (composite of heterogeneous treatment modalities; short follow‐up in most studies) Very serious imprecision (*n* = 13 total events; most studies did not report systematically)	**⊕**○○○ **VERY LOW**

## 4 Discussion

This systematic review and structured quantitative synthesis is the first to synthesise evidence specifically addressing EWDR following HCL initiation in T1D, drawing on eight studies encompassing 1487 individuals. Across single‐arm HCL cohorts, study‐defined EWDR was reported in 8.9% to 27.6% of participants, and in 8.9% to 26.5% of participants among studies with follow‐up ≤ 12 months. These estimates overlap the 13.1% EWDR rate documented in the intensive treatment arm of the DCCT [5]. Comparative studies were directionally inconsistent, with study‐specific risk ratios ranging from 0.32 to 1.51. Included studies differed substantially in EWDR definition, retinal imaging and grading methods, follow‐up duration and comparator therapy. Pooled summary estimates were not presented; the current evidence base is too heterogeneous and methodologically limited to quantify risk reliably.

The largest and methodologically strongest prospective study in this review, Nattero‐Chávez et al. [15] deserves specific discussion, because, while it reported retinopathy outcomes in a HCL cohort, it is not, in the strictest sense, a study of EWDR. With a mean follow‐up of 4.9 years and annual retinal assessments, its design is optimally suited to evaluating the medium‐ to long‐term impact of HCL on DR incidence and progression, not the short‐term paradoxical worsening that occurs in the first months after glycaemic intensification. The study demonstrated a 67.2% reduction in DR risk in the HCL versus MDI group, with all DR progression events occurring in the MDI group, a finding highly consistent with the DCCT's long‐term message that intensive glycaemic control is profoundly retino‐protective despite any short‐term early worsening [3, 22]. Notably, the study also identified that longitudinal increases in HbA1c during follow‐up, rather than the initial reduction, were associated with greater DR risk, reinforcing that sustained glycaemic improvement is the dominant long‐term determinant of retinal outcomes. These results provide important reassurance that HCL use is unlikely to confer long‐term retinal harm. However, because many participants were already established on HCL at study entry, with a mean HCL exposure at baseline of 10.7 months, the study population had, in large part, already passed through the highest‐risk EWDR window before observation began. It therefore cannot exclude an early‐worsening signal in the first months after HCL initiation and cannot be used to directly estimate EWDR incidence in the way that shorter‐term studies with assessments anchored to HCL start can. A methodological point warrants emphasis regarding our handling of the Nattero‐Chávez et al. [15] data. Although the authors reported no worsening of DR in the HCL group, we elected to include this incident rate within our synthesis because, conceptually, the development of new‐onset DR is itself a form of retinal worsening that overlaps with how EWDR was defined elsewhere. Several of the included studies defined worsening as any rise in retinopathy grade, which necessarily captures transitions from no retinopathy to minimal or background disease, in effect, incident DR by another name. Including the Nattero‐Chávez incident rate therefore aligns the outcomes used by the other studies and avoids artificially excluding a relevant cohort on definitional grounds alone.

A clinically important and counterintuitive finding of this review is the inconsistency of HbA1c reduction as a predictor of EWDR in the HCL context. In the DCCT and subsequent pre‐HCL intensification literature, the magnitude of HbA1c fall was established as a primary determinant of EWDR risk [4, 23, 24]. However, across the studies included in this review, only Diddhenipothage et al. [17] identified a clear association between the degree of HbA1c reduction (≥ 2%) and EWDR; two studies (Johansson et al. and Karakus et al.) found no significant relationship between HbA1c improvement and worsening. One interpretation is that the mechanism of glycaemic improvement in HCL differs sufficiently from the intensive MDI regimens studied in the DCCT that the same change in HbA1c no longer reliably predicts retinal risk. Under this hypothesis, it is the rate and pattern of glucose change, rather than the endpoint HbA1c reduction, that drives the haemodynamic and growth factor‐mediated mechanisms of EWDR [25]. HCL differs from the intensive multiple‐injection regimens of the DCCT era not only in the speed of glycaemic improvement but in the quality of the resulting control. HCL produces markedly lower glycaemic variability and substantially less hypoglycaemia than conventional intensification. Neither feature is captured by HbA1c, yet both could be plausible modifiers of retinal risk, and the near absence of significant hypoglycaemia with HCL may attenuate some mechanisms implicated in retinal injury. This reinforces the need for CGM derived metrics to be captured and pre‐specified as candidate predictors in future studies, rather than relying on HbA1c change alone. An alternative interpretation is that the included studies were underpowered to detect a HbA1c‐reduction effect; most had fewer than 200 participants and were not designed to test this specific association. If crude HbA1c reduction is not a reliable risk predictor in the HCL context, then clinicians cannot use standard pre‐HCL era risk stratification frameworks to identify which patients require enhanced ophthalmological monitoring. The finding that baseline HbA1c, rather than its rate of change, was the more consistently identified predictor across three studies may therefore be the more relevant clinical signal. In parallel, systematic review evidence on fibrate therapy supports a wider concept that retinal outcomes in diabetes may be modified by systemic metabolic therapies beyond just glycaemic control [26].

The most fundamental methodological limitation of the current evidence is that six of eight included studies were retrospective, with retinal outcome data extracted from routine clinical records rather than collected under a protocol‐specified surveillance schedule. This has direct implications for the validity of EWDR estimates. EWDR is a time‐sensitive phenomenon, with the highest‐risk window established from DCCT data as occurring within the first 3–12 months of glycaemic intensification [5]. Retrospective extraction of retinal gradings from clinical records means that the interval between HCL initiation and retinal assessment was not standardised and, in several studies, was highly variable both within and between participants. Karakus et al. [19] acknowledged that retinal assessments were performed at any point from 3 months to more than 2 years after HCL initiation, a window that spans the EWDR risk period and extends beyond it into the phase of natural longitudinal DR progression. Under these conditions, observed DR changes cannot be confidently attributed to the acute glycaemic improvement associated with HCL initiation, as distinct from background retinopathy progression that would have occurred regardless of HCL use. No study included in this review used a pre‐specified, protocol‐driven retinal surveillance schedule anchored to the date of HCL initiation.

A further source of heterogeneity was the inconsistency in how EWDR was defined and graded across studies. Some studies defined EWDR exclusively as worsening of a pre‐existing DR grade, excluding individuals who developed new incident DR during follow‐up; others included both incident and progressive events within a single composite; and others used qualitative grading without specifying explicit thresholds for what constituted meaningful change. Additionally, the grading systems used varied across national contexts which are not equivalent and use different thresholds for defining clinically meaningful change. Together, these differences limit the direct interpretability of between‐study comparisons and contribute to the substantial statistical heterogeneity limiting reporting of pooled results through meta‐analyses.

The strengths of this review include its pre‐registered protocol, comprehensive multi‐database search with no language restriction, dual‐reviewer screening and data extraction, and transparent risk of bias assessment using ROBINS‐I. It is, to our knowledge, the first systematic review to synthesise evidence specifically on EWDR in the context of HCL initiation. The inclusion of studies where retinopathy was a secondary or incidental endpoint ensured capture of all available relevant data and avoided the bias of including only studies that set out to examine this question. The principal limitations of this review include the retrospective design of six of eight included studies, the absence of protocol‐specified retinal surveillance anchored to HCL initiation, the heterogeneity of EWDR definitions and grading systems, and the small number of eligible studies (*n* = 8). These limitations precluded reliable pooled estimation and mean that the study‐level rates reported here should be interpreted as descriptive signals rather than precise incidence estimates. All included studies were observational and the majority retrospective, and because confounding by baseline HbA1c, age, diabetes duration, baseline DR severity, prior treatment, and selection into HCL therapy cannot be excluded, no causal conclusion can be drawn regarding HCL initiation and EWDR risk. Applying a GRADE‐based appraisal, the certainty of evidence was very low for all outcomes.

The evidence base is geographically concentrated in Europe, Australasia, and the USA, which may limit generalisability to populations with different healthcare access, HCL initiation pathways, or background diabetic eye disease prevalence. An additional unresolved question is whether study‐defined EWDR after HCL initiation translates into clinically meaningful visual outcomes. Most included studies reported changes in retinopathy grade and incident DR, but did not systematically assess visual acuity, patient‐reported visual function, or the persistence and reversibility of retinal changes. As a result, the current literature cannot determine whether retinal worsening observed after HCL initiation is transient and subclinical, or whether it carries meaningful implications for vision, quality of life, or ophthalmic treatment burden. This distinction between retinal grade worsening and clinically meaningful ophthalmic endpoints is important. Treatment‐requiring disease represents a later and more clinically consequential stage than minor change in grade alone [27].

Finally, the absence of CGM‐derived metrics (TIR, glycaemic variability etc.) as reported predictors in most included studies means that the question of whether change in TIR/TAR/GV is a better predictor of EWDR than the change in HbA1c remains unanswered.

The most urgent research need identified by this review is a prospective study with protocol‐specified retinal assessments anchored to the time of HCL initiation. Such studies should include retinal imaging at baseline and pre‐defined early follow‐up intervals, apply a standardised validated grading system, incorporate measures of retinal structure and function, and pre‐specify analyses of CGM‐derived glycaemic metrics. These priorities directly inform the rationale for the INSIGHT‐1 study (ISRCTN11740459 [28]), a prospective observational study evaluating retinal outcomes in adults with T1D initiating HCL therapy, with protocol‐specified multimodal ophthalmic assessments anchored to HCL initiation. Beyond estimating incidence, prospective datasets of this kind will be essential for developing and validating a clinical risk score to identify individuals who may require enhanced ophthalmological surveillance at HCL initiation: incorporating age, diabetes duration, baseline HbA1c, baseline DR grade, prior insulin delivery modality, and early CGM‐derived glycaemic change.

## 5 Conclusion

This systematic review provides the first synthesis of evidence on EWDR following HCL therapy in T1D. Study‐defined retinal worsening was reported in a minority of HCL studies, and substantial clinical and methodological heterogeneity precluded reliable pooled estimation and meta‐analysis. The current evidence base is dominated by retrospective studies, variable retinal assessment timing and inconsistent EWDR definitions, and is of very low certainty; it does not permit any causal inference regarding HCL initiation and EWDR risk. Well‐designed prospective studies with protocol‐specified retinal surveillance anchored to HCL initiation are required to generate reliable incidence estimates, identify risk factors, determine visual consequences, and inform standardised screening guidance.

## Funding

This work was supported by Breakthrough T1D (previously known as JDRF) and the Novo Nordisk UK Research Foundation.

## Conflicts of Interest

M.A. receives a fellowship from the Novo Nordisk UK research foundation and Breakthrough T1D and collaborative funding from Procter & Gamble. U.A. has received honoraria from Eli Lilly, Procter & Gamble, Viatris, Grunenthal, Theras and Sanofi for educational meetings and funding for attendance to an educational meeting from Diiachi Sankyo. U.A. has also received investigator‐led funding by Procter & Gamble, GSK and Axonis Therapeutics and is a council member of the Royal Society of Medicine's Vascular, Lipid & Metabolic Medicine Section. U.A. is supported by the National Institute for Health Research Applied Research Collaboration North West Coast (ARC NWC). P.I.B. has received honoraria from Roche, Bayer, Alimera and AbbVie and research funding from Roche. The views expressed in this publication are those of the author(s) and not necessarily those of the National Institute for Health Research or the Department of Health and Social Care. AI has received investigator‐led funding from Dexcom, Abbott and has received honoraria from Eli Lilly, Sanofi, Boehringer Ingleheim and Diiachi Sankyo as speaker fees.

## Supporting information


**Data S1:** Graphical abstract.

**Data S2:** Search strategy.

## Data Availability

Data is publicly available.

## References

[1] T. Y. Wong , C. M. G. Cheung , M. Larsen , S. Sharma , and R. Simó , “Diabetic Retinopathy,” Nature Reviews. Disease Primers 2 (2016): 16012. 10.1038/nrdp.2016.12 27159554

[2] J. W. Y. Yau , S. L. Rogers , R. Kawasaki , et al., “Global Prevalence and Major Risk Factors of Diabetic Retinopathy,” Diabetes Care 35 (2012): 556–564. 22301125 10.2337/dc11-1909 PMC3322721

[3] The Diabetes Control and Complications Trial Research Group , “The Effect of Intensive Treatment of Diabetes on the Development and Progression of Long‐Term Complications in Insulin‐Dependent Diabetes Mellitus,” New England Journal of Medicine 329 (1993): 977–986. 8366922 10.1056/NEJM199309303291401

[4] K. Dahl‐Jørgensen , O. Brinchmann‐Hansen , K. F. Hanssen , L. Sandvik , and O. Aagenaes , “Rapid Tightening of Blood Glucose Control Leads to Transient Deterioration of Retinopathy in Insulin Dependent Diabetes Mellitus: The Oslo Study,” BMJ 290 (1985): 811–815. 3919804 10.1136/bmj.290.6471.811 PMC1418598

[5] The Diabetes Control and Complications Trial Research Group , “Early Worsening of Diabetic Retinopathy in the Diabetes Control and Complications Trial,” Archives of Ophthalmology 116 (1998): 874. 9682700 10.1001/archopht.116.7.874

[6] S. Feldman‐Billard , É. Larger , and P. Massin , “Early Worsening of Diabetic Retinopathy After Rapid Improvement of Blood Glucose Control in Patients With Diabetes,” Diabetes & Metabolism 44 (2018): 4–14. 29217386 10.1016/j.diabet.2017.10.014

[7] H. Akil , J. Burgess , S. Nevitt , S. P. Harding , U. Alam , and P. Burgess , “Early Worsening of Retinopathy in Type 1 and Type 2 Diabetes After Rapid Improvement in Glycaemic Control: A Systematic Review,” Diabetes Therapy 13 (2022): 1–23. 10.1007/s13300-021-01190-z PMC8776958 34928488

[8] E. G. Wilmot , J. Beltrand , B. Guerci , et al., “Tubeless Automated Insulin Delivery Versus Multiple Daily Injections in Children and Adults With Type 1 Diabetes With Elevated HbA1c (RADIANT): A Multicentre, International, Parallel‐Group, Open‐Label, Randomised, Controlled Trial,” Lancet Diabetes and Endocrinology 14 (2026): 305–316. 41747751 10.1016/S2213-8587(25)00364-X

[9] T. S. J. Crabtree , T. P. Griffin , Y. W. Yap , et al., “Hybrid Closed‐Loop Therapy in Adults With Type 1 Diabetes and Above‐Target HbA1c: A Real‐World Observational Study,” Diabetes Care 46 (2023): 1831–1838. 37566697 10.2337/dc23-0635 PMC10516256

[10] A. L. Liarakos , T. S. J. Crabtree , T. P. Griffin , et al., “Hybrid Closed‐Loop Therapy in Adults With Type 1 Diabetes in England: Long‐Term Outcomes From a Real‐World Observational Study,” Diabetes Technology & Therapeutics 27 (2025): 820–830. 40445741 10.1089/dia.2025.0165 PMC7618701

[11] M. Phillip , R. Nimri , R. M. Bergenstal , et al., “Consensus Recommendations for the Use of Automated Insulin Delivery Technologies in Clinical Practice,” Endocrine Reviews 44 (2023): 254–280. 36066457 10.1210/endrev/bnac022 PMC9985411

[12] M. Bajaj , R. G. McCoy , K. Balapattabi , et al., “12. Retinopathy, Neuropathy, and Foot Care: Standards of Care in Diabetes—2026,” Diabetes Care 49 (2026): S261–S276. 41358886 10.2337/dc26-S012 PMC12690177

[13] M. J. Page , J. E. McKenzie , P. M. Bossuyt , et al., “The PRISMA 2020 Statement: An Updated Guideline for Reporting Systematic Reviews,” BMJ 372 (2021): n71. 33782057 10.1136/bmj.n71 PMC8005924

[14] J. A. Sterne , M. A. Hernán , B. C. Reeves , et al., “ROBINS‐I: A Tool for Assessing Risk of Bias in Non‐Randomised Studies of Interventions,” BMJ 355 (2016): i4919. 27733354 10.1136/bmj.i4919 PMC5062054

[15] L. Nattero‐Chávez , S. de Lope Quiñones , A. Quintero Tobar , et al., “Automated Insulin Delivery Reduces the Risk of Diabetic Retinopathy in Adults With Type 1 Diabetes: A Prospective Cohort Study,” Diabetes Technology & Therapeutics 28, no. 7 (2026), 10.1177/15209156251411929. 41645537

[16] B. Barquiel , D. Álvarez , Ó. M. Domínguez , et al., “Diabetic Retinopathy Remission in Patients Using an Automated Insulin Delivery System: A Prospective Controlled Study,” Diabetes & Metabolism 51 (2025): 101703. 40947051 10.1016/j.diabet.2025.101703

[17] S. A. D. M. Diddhenipothage , “Emerging Risk Factors and Treatment Options for Diabetic Eye Disease,” in EASD Madrid Oral Presentations (EASD, 2024), https://www.easd.org/media‐centre/#!resources/b‐is‐early‐worsening‐of‐diabetic‐retinopathy‐a‐concern‐when‐starting‐hybrid‐closed‐loop‐therapy‐in‐people‐with‐type‐1‐diabetes‐b.

[18] R. Andrew , T. Crabtree , P. Richardson , et al., “Poster Abstracts,” Diabetic Medicine 40, no. S1 (2023), 10.1111/dme.15048.

[19] K. E. Karakus , H. K. Akturk , J. K. Snell‐Bergeon , and V. N. Shah , “Progression of Diabetic Retinopathy After Initiation of Automated Insulin Delivery System in Adults With Type 1 Diabetes,” Journal of Diabetes Science and Technology 20, no. 4 (2025), 10.1177/19322968251318740. PMC11831613 39950376

[20] M. Ung , A. Penfornis , C. Valentim , S. Franc , C. Amadou , and J. Eroukhmanoff , “Hybrid Closed Loop in Adolescents and Young Adults Living With Type 1 Diabetes: A Real‐World Study,” Diabetes Technology & Therapeutics 27 (2025): 760–764. 40208827 10.1089/dia.2024.0528

[21] M. M. E. Johansson , F. March de Ribot , M.‐J. Sime , et al., “Short‐Term Diabetic Retinopathy Status in People With Type 1 Diabetes Commencing Automated Insulin Delivery,” Diabetes Technology & Therapeutics 27 (2025): 386–394. 39925093 10.1089/dia.2024.0568

[22] Action to Control Cardiovascular Risk in Diabetes Follow‐On (ACCORDION) Eye Study Group and the Action to Control Cardiovascular Risk in Diabetes Follow‐On (ACCORDION) Study Group , “Persistent Effects of Intensive Glycemic Control on Retinopathy in Type 2 Diabetes in the Action to Control Cardiovascular Risk in Diabetes (ACCORD) Follow‐On Study,” Diabetes Care 39 (2016): 1089–1100. 27289122 10.2337/dc16-0024 PMC4915557

[23] The Kroc Collaborative Study Group , “Diabetic Retinopathy After Two Years of Intensified Insulin Treatment,” JAMA 260 (1988): 37. 2898025 10.1001/jama.1988.03410010045032

[24] E. van Ballegooie , J. M. M. Hooymans , Z. Timmerman , et al., “Rapid Deterioration of Diabetic Retinopathy During Treatment With Continuous Subcutaneous Insulin Infusion,” Diabetes Care 7 (1984): 236–242. 6734393 10.2337/diacare.7.3.236

[25] E. Chantelau , “Evidence That Upregulation of Serum IGF‐1 Concentration Can Trigger Acceleration of Diabetic Retinopathy,” British Journal of Ophthalmology 82 (1998): 725–730. 9924360 10.1136/bjo.82.7.725 PMC1722687

[26] K.‐Y. Chen , H.‐C. Chan , and C.‐M. Chan , “Can Fibrate Therapy Redefine the Management of Diabetic Retinopathy? A Comprehensive Systematic Review and Meta‐Analysis of Efficacy and Safety,” Journal of Diabetes and Its Complications 39 (2025): 109178. 41038126 10.1016/j.jdiacomp.2025.109178

[27] K.‐Y. Chen , H.‐C. Chan , and C.‐M. Chan , “Which Treatment Modality Offers the Best Outcomes for Diabetic Retinopathy? A Systematic Review and Network Meta‐Analysis,” Diabetes Research and Clinical Practice 227 (2025): 112379. 40701389 10.1016/j.diabres.2025.112379

[28] M. Anson and U. Alam , Hybrid Closed Loop Insulin Pumps and Early Worsening of Diabetic Retinopathy in Type 1 Diabetes, 2025, https://www.isrctn.com/ISRCTN11740459.

