# Supplementary material for: Early Worsening of Diabetic Retinopathy Following Initiation of Hybrid Closed‐Loop/Automated Insulin Delivery Systems in Type 1 Diabetes: A Systematic Review and Structured Study‐Level Synthesis
Source: Diabetes Obes Metab. 2026 Jul 8;28(10):8878–94. doi: 10.1111/dom.71084 (PMC13538795; doi:10.1111/dom.71084)
Supplement: Supplementary file 1 [file DOM-28-8878-s001.docx]

**SCOPUS**

TITLE-ABS-KEY ( "type 1 diabetes" OR "type I diabetes" OR T1D OR T1DM OR "insulin-dependent diabetes" OR "insulin dependent diabetes" OR "juvenile diabetes" ) AND TITLE-ABS-KEY ( "diabetic retinopathy" OR retinopath* OR maculopath* OR "diabetic macular oedema" OR "diabetic macular edema" OR DMO OR DME OR "proliferative diabetic retinopathy" OR PDR OR "non-proliferative diabetic retinopathy" OR NPDR OR "early worsening" OR EWDR OR "retinopathy progression" OR "retinopathy worsening" OR "retinal progression" OR "retinal worsening" OR "fundus photograph*" OR "fundal photograph*" OR "retinal photograph*" OR "retinal imaging" ) AND TITLE-ABS-KEY ( "automated insulin delivery" OR "hybrid closed loop" OR "hybrid closed-loop" OR HCL OR "closed loop insulin" OR "closed-loop insulin" OR "closed loop therapy" OR "closed-loop therapy" OR "artificial pancreas" OR "algorithm-driven insulin" OR "automated basal insulin" OR "automated correction" OR "Control-IQ" OR "Control IQ" OR "MiniMed 780G" OR 780G OR "Omnipod 5" OR OMP5 OR OP5 OR CamAPS OR "CamAPS FX" OR Diabeloop OR DBLG1 OR Loop OR AndroidAPS OR iAPS OR ( "insulin pump*" W/3 ( automated OR algorithm* OR "closed loop" OR "closed-loop" OR hybrid ) ) OR ( CSII W/3 ( automated OR algorithm* OR "closed loop" OR "closed-loop" OR hybrid ) ) )

**Web of Science**

TS=(
("type 1 diabetes" OR "type I diabetes" OR T1D OR T1DM OR "insulin-dependent diabetes" OR "insulin dependent diabetes" OR "juvenile diabetes")
AND
("diabetic retinopathy" OR retinopath* OR maculopath* OR "diabetic macular oedema" OR "diabetic macular edema" OR DMO OR DME OR
"proliferative diabetic retinopathy" OR PDR OR "non-proliferative diabetic retinopathy" OR NPDR OR
"early worsening" OR EWDR OR "retinopathy progression" OR "retinopathy worsening" OR "retinal progression" OR "retinal worsening" OR
"fundus photograph*" OR "fundal photograph*" OR "retinal photograph*" OR "retinal imaging" OR "eye screening")
AND
("automated insulin delivery" OR "hybrid closed loop" OR "hybrid closed-loop" OR HCL OR
"closed loop insulin" OR "closed-loop insulin" OR "closed loop therapy" OR "closed-loop therapy" OR
"artificial pancreas" OR "algorithm-driven insulin" OR "automated basal insulin" OR "automated correction" OR
"automated insulin pump*" OR "algorithm-driven insulin pump*" OR
"Control-IQ" OR "Control IQ" OR "MiniMed 670G" OR "MiniMed 780G" OR 670G OR 780G OR
"Omnipod 5" OR OMP5 OR OP5 OR CamAPS OR "CamAPS FX" OR Diabeloop OR DBLG1 OR AndroidAPS OR iAPS)
)

**MEDLINE**

| **Line** | **Search strategy** | **Results** |
| --- | --- | --- |
| 1 | exp Diabetes Mellitus, Type 1/ | 93,971 |
| 2 | exp Diabetic Retinopathy/ | 33,486 |
| 3 | exp Macular Edema/ | 10,112 |
| 4 | (diabetic retinopath* or retinopath* or maculopath* or diabetic macular oedema or diabetic macular edema or DMO or DME or proliferative diabetic retinopath* or PDR or non-proliferative diabetic retinopath* or NPDR).mp. | 91,709 |
| 5 | (early worsen* or EWDR or retinopath* progress* or retinopath* worsen* or retinal worsen* or retinal progress*).mp. | 635 |
| 6 | (fundus photograph* or fundal photograph* or retinal photograph* or retinal imaging or ophthalmolog* assessment or eye screen*).mp. | 14,565 |
| 7 | 2 or 3 or 4 or 5 or 6 | 105,738 |
| 8 | exp Insulin Infusion Systems/ | 7,402 |
| 9 | exp Pancreas, Artificial/ | 1,109 |
| 10 | (automated insulin deliver* or AID or hybrid closed loop* or HCL or closed loop insulin or closed-loop insulin or closed loop therap* or closed-loop therap* or artificial pancreas or algorithm-driven insulin or automated basal insulin or automated correction*).mp. | 303,575 |
| 11 | (Control-IQ or "Control IQ" or MiniMed 780G or 780G or CamAPS or CamAPS FX or Diabeloop or DBLG1 or Loop or AndroidAPS or iAPS or OMPS or Omnipod 5 or OP5).mp. | 214,740 |
| 12 | (insulin pump* or continuous subcutaneous insulin infusion or CSII or automated or algorithm* or closed-loop or closed loop or hybrid).mp. | 1,105,786 |
| 13 | 8 or 9 or 10 or 11 or 12 | 1,576,888 |
| 14 | 1 and 7 and 13 | 319 |
